# Special Issue “COVID-19 and Thrombosis”

**DOI:** 10.3390/v14071425

**Published:** 2022-06-29

**Authors:** Pierpaolo Di Micco, Egidio Imbalzano, Giuseppe Camporese

**Affiliations:** 1 Uoc Medicina, P.O. A. Rizzoli–Asl Na2 Nord, Lacco Ameno, 80076 Naples, Italy; 2 General Medicinem, Thrombotic and Haemorragic Unit, Department of Internal Medicine, University of Messina, 98122 Messina, Italy; egidio.imbalzano@unime.it; 3 Department of Medicine, University of Padua, 35122 Padua, Italy; giuseppe.camporese@aopd.veneto.it

Since the pandemic began, an association among COVID-19 and venous thromboembolism has been reported, in particular for inpatients.

This association covers several items from pathophysiology to prognosis: from interactions between SARS-CoV-2 and respiratory cells by heparin/heparan sulphate, endothelial dysfunction, and prothrombotic cytokine storm to finding methods to perform early diagnosis of VTE in inpatients with COVID-19 and finding the right drugs to prevent and to treat VTE in patients with COVID-19.

The interaction between SARS-CoV-2, heparan sulphate, and ACE receptors 1 and 2 were described early on, and these interactions were reported as main actors of the following induced hypercoagulable state [1]. Furthermore, additional prothrombotic conditions were found between comorbidities and induced hypomobility for intensive or sub-intensive hospital care.

Endothelial dysfunction due to the respiratory damages and virally induced inflammation is responsible for the abnormal release of vWF [2]. Furthermore, endothelial dysfunctions are also able to induce other prothrombotic action because of platelets’ activation and the release of other molecules, such as cadherins [3].

Endothelial dysfunctions remain for a long time due to cytokine storm, and this abnormality may induce a persistent prothrombotic state. Therefore, many other transient risk factors may induce further pathophysiological changes that are associated with a worsening prognosis [4,5,6,7].

Moreover, for these types of dysfunctions, the protective role of heparins was testified in inpatients with COVID-19, where it crossed its anti-thrombotic actions and took anti-viral and anti-inflammatory roles [1,8]. For this reason, a really complex clinical debate has been taken place for several months on the right prophylactic or therapeutic dosage of heparins in inpatients with COVID-19 [9,10,11].

On the other hand, prothrombotic conditions may also trigger thrombotic diseases different from VTE during COVID-19: atherothrombotic diseases such as coronary heart disease increased their incidence during infection by SARS-CoV-2, as well as other thrombotic diseases of small vessels [12].

From a clinical point of view, such as complex scenario associated with the use of antithrombotic drugs at different posologies is also associated with clinical bleedings [13].

Furthermore, as a leitmotif, thrombosis has also been described as the most dangerous complication of the COVID-19 vaccination campaign. VITT was reported for all types of anti-SARS-CoV-2 vaccines, and its pathophysiology and prevention have been debated for a long time [14,15]. However, cerebral vein thrombosis has not been the only type of venous thrombosis detected after vaccination, as reported in a large registry [16]

In summary, we can conclude that after 2 years of this pandemic and several studies on its pathophysiology and clinical thrombotic disease, some viral infections, such as COVID-19, are able to induce an associated life-threatening, pro-thrombotic condition as well as bacterial infection by mechanisms that are associated with prolonged inflammation. These experiences may play an important role in the field of prevention when other outbreaks occur.
